# Association of School‐Based Health Center Availability With Child Mental Health Outcomes

**DOI:** 10.1111/1475-6773.70042

**Published:** 2025-09-11

**Authors:** Carrie E. Fry, Mason Shero, Melinda B. Buntin, Carolyn J. Heinrich

**Affiliations:** ^1^ Assistant Professor, Department of Health Policy Vanderbilt University School of Medicine Nashville USA; ^2^ Assistant Professor Denison College Granville USA; ^3^ Bloomberg Distinguished Professor, Bloomberg School of Public Health and Carey Business School Johns Hopkins University Washington USA; ^4^ Patricia and Rodes Hart Professor of Education, Public Policy, and Economics, Department of Leadership, Policy, and Organizations Vanderbilt University Nashville Tennessee USA

## Abstract

**Objective:**

To estimate changes in student mental health outcomes after the adoption of a school‐based health center (SBHC).

**Study Setting/Design:**

Using a retrospective, quasi‐experimental design, this study compared changes in mental health diagnoses and healthcare utilization among students in school districts that adopted an SBHC to students in districts that did not adopt an SBHC, before and after adoption. A stacked difference‐in‐differences estimator was used to address the staggered adoption of SBHCs and the potential for heterogeneous treatment effects. Health conditions (measured via diagnosis codes) and health care use (measured via procedure codes and place‐of‐service codes) were obtained from Medicaid inpatient, outpatient, physician, and pharmacy claims.

**Data Sources and Analytic Sample:**

Information on the availability of SBHCs was obtained via census of 142 of Tennessee's 147 public school districts. Using secondary data from administrative health and education records, we probabilistically linked Tennessee students enrolled in Medicaid to public‐school records from 2006 to 2021. We linked approximately 70% of students enrolled in a Tennessee public school to Medicaid records.

**Principal Findings:**

We identified 41 districts with an SBHC between 2007 and 2019. After the adoption of an SBHC, districts with an SBHC had a 0.5 (95% CI: −0.9, −0.2) percentage point decline in the proportion of students with any mental health diagnosis, which corresponds to a 6.6% relative decline. This was driven by a decrease in the diagnosis of depression, anxiety, and attention deficit and hyperactivity disorder (ADHD). We also found a significant increase in outpatient mental health care visits and a decrease in emergency department visits for mental health conditions after the adoption of an SBHC.

**Conclusions:**

SBHCs are one mechanism through which the mental health needs of school‐aged children are met. Timely and adequate resources are needed to ensure SBHCs can sustain their services in this time of need.

**Summary**

- What is known?
  ○ Mental health conditions are on the rise among American youth.
  ○ School‐based health centers (SBHCs), which provide comprehensive physical and mental health care, have proliferated over the past two decades.
  ○ SBHCs are associated with improvements in self‐reported health and some physical health conditions, but evidence on mental health is limited and mixed.
- What this study adds?
  ○ This study is one of the first to examine the role of SBHCs on mental health diagnoses and healthcare use using a census‐based approach to SBHC identification in Tennessee.
  ○ SBHCs are associated with reductions in mental health diagnoses, emergency department use, and medication use for mental health conditions and increases in school‐based visits and outpatient mental health visits.
  ○ SBHCs in Tennessee may reduce the symptom burden of mental health conditions and link students with appropriate outpatient mental healthcare.

## 1 Introduction

Estimates from the late 2010s suggest that one in five children and adolescents is diagnosed with a mental health condition each year, and two in five adolescents will meet the criteria for a mental health condition by age 18 [1]. Moreover, rates of diagnosed depression and anxiety among adolescents increased by more than 25% between 2016 and 2020 [1], and rates of ED use increased 60%, 159%, and 329% for mental health, substance use, and deliberate self‐harm, respectively [2].

Alarmingly, an estimated 35%–50% of children with a mental health or substance use disorder do not receive treatment in a year [3]. With the closure of schools during the pandemic, access to and utilization of school‐based primary care and treatment for behavioral health conditions worsened for adolescents [4].

Schools play an important role in identifying and attending to children's mental health needs [5]. They serve as the most common institutional entry point to mental health services for youth and often play a key role in connecting them with services to improve health and educational outcomes [6]. More than half of all adolescents who received mental health services accessed them in an educational setting, either alone or in conjunction with a non‐educational setting, and those who accessed mental health services in an educational setting *only* were more likely to be from low‐income households, publicly insured, or identified as a racial or ethnic minority [7].

School‐based health centers (SBHCs) represent one way that schools may provide healthcare services to their students, faculty, staff, and sometimes community members. SBHCs are staffed by trained medical professionals and provide comprehensive health care to children within the school environment. Services may include but are not limited to well‐child visits, sports physicals, care for chronic conditions (e.g., Type I diabetes, asthma, attention deficit and hyperactivity disorder [ADHD]), sick visits, and vaccinations) [8]. Increasingly, SBHCs also provide screening and treatment for mental health conditions. Recent estimates suggest that there are more than 2500 SBHCs across the U.S. that provide access to care for 8% to 10% of public‐school students [9, 10], with more SBHCs in areas with higher proportions of low‐income and underserved children, such as those who live in rural areas [11].

However, previous research on SBHC effectiveness in improving the mental health outcomes of children produced mixed evidence with limitations of internal validity [11, 12, 13, 14, 15, 16, 17]. A recent review identified 11 studies that examined the relationship between SBHCs and mental health utilization; these studies span data from 1989 to 2009 [18]. Of these studies, seven examine the role of SBHCs in access to and use of mental health care. All seven found increases in access to care and utilization among students who have access to an SBHC but lack methodological rigor with no comparison group and/or pre‐SBHC data only.

Another review of SBHC's effects on child health and education outcomes identified only one study that examined mental health outcomes, which found that students who had access to mental health services through an SBHC were less likely to self‐report depressive episodes or suicidal thoughts and behaviors [11, 19]. One other paper identified uses pre‐post data in both districts with and without an SBHC to examine SBHCs' role in improving outcomes for students with mental health conditions [20]. However, this study includes data from 1997 to 2003 and does not use a quasi‐experimental design (like a difference‐in‐differences) to estimate the differential changes of outcomes in SBHC districts compared to non‐SBHC districts. Given the limitations of previous work, recent research has highlighted the need for more rigorous, empirical work on the role of schools in child mental health [21].

We expand on this evidence base by using recent variation over time and across districts in SBHC adoption in Tennessee. This study is among the first to include both pre‐SBHC data and a comparator (districts without an SBHC) with mental health outcomes and healthcare use among a general student population. Additionally, our study uses more recent data (2006–2021), reflecting a period of increased mental health needs of children and adolescents, and administrative data on both mental health outcomes and healthcare use to explore the mechanisms through which SBHCs operate.

## 2 Data and Methods

We used the Andersen Behavioral Model of Health Service Use to guide our analytic approach for this study [22]. The Andersen Model identifies the predisposing, enabling, and need‐based factors that affect health care use. In the following sections and Figure S1, we identify and operationalize each of these factors and outcomes.

### 2.1 Data Sources

We used longitudinal, linked data including health and education outcomes of school‐aged children in Tennessee between 2006 and 2021. Health outcomes were derived from insurance claims from Tennessee's Medicaid program, TennCare. Data from the Tennessee Department of Education provide demographic characteristics and identify the schools in which children were enrolled.

We linked students' education and health data through a probabilistic matching algorithm that included name, social security number, and date of birth [23]. Children were included in the dataset if they were enrolled in TennCare and a public school in a given academic year, totaling 1,575,411 unique children and 9,705,840 student‐year observations. Our linked data represent 67.5% of students in Tennessee enrolled in a public school between 2006 and 2021. Data were aggregated up to and analyzed at the school‐academic year level.

### 2.2 Student Characteristics (Covariates)

Student‐level characteristics (racial/ethnic identity; sex; special education needs; being an English language learner) were considered predisposing characteristics affecting students' accessing health care. Predisposing characteristics are largely immutable but predict one's health status and access to care. As an example, previous evidence suggests that students from different racial/ethnic backgrounds have differential access to care and attend schools with differing levels of health and education resources [24, 25]. We also included several student‐level characteristics that are enabling factors –housing, poverty, and immigration status–as covariates that capture barriers to receiving care [26, 27].

Both predisposing and enabling factors were included in our multivariate regression models as covariates. Differences in the ‘levels’ of a covariate across groups are not necessarily confounders in a difference‐in‐differences (DID) analysis; these level differences must change by group over time to confound the relationship between SBHCs and mental health outcomes. If the effect of racial identity on mental health changes over time differentially in districts with SBHCs compared to those without, then the racial composition of a student body could act as a confounder in this analysis [28, 29]. Given that enabling factors are more mutable than predisposing factors, these factors may change in response to the implementation of an SBHC, acting as confounders in a DID analysis that need to be appropriately accounted for in regression models.

Because our analysis is at the school‐academic year level, we aggregated student characteristics to school‐academic year averages and used these averages as covariates in our DID regression. The operationalization of these characteristics is described in Table S1.

### 2.3 Independent Variable

The independent variable is the adoption of a SBHC. We identified school districts in Tennessee with an SBHC (defined as school‐based, school‐linked, telehealth or mobile health) by contacting each of the 147 districts and inquiring about the presence of an SBHC and the years the SBHC was operational. We confirmed the SBHC status and operational dates for 142 (97%) of these school districts. While some SBHCs may be physically located in specific schools within a district, SBHCs and their services were available to any student attending a school in the district. In other districts, SBHCs may be linked to all schools in the district, or they may be mobile and regularly visit each school in the district. For these reasons, we assign treatment status at the district level. In other words, we assume that each school in a district is equally affected by the adoption of an SBHC. However, we conduct our analysis at the school‐academic year level, which allows us to directly adjust for the age distribution of students in a school because some conditions and healthcare use may be more prevalent in older students compared to younger students. Finally, we only included SBHCs identified through 2019 in the treated group because of the effect the pandemic likely had on SBHC adoption and access to healthcare services through an SBHC. We hypothesized that SBHCs also act as enabling factors—students that have access to them are more likely to engage in care than students without adequate access to them.

### 2.4 Outcomes of Interest

The outcomes of interest for this study are behavioral health diagnoses and health care use. Diagnoses are measures of evaluated need in the Anderson model framework and may drive health care use or other health behaviors. We examined diagnoses of specific health conditions derived from Medicaid inpatient, outpatient, and pharmacy claims data. We included a composite measure of any mental health condition and specific conditions. The composite measure included diagnoses of ADHD, anxiety, bipolar disorder, depression, eating disorders, oppositional defiant and other conduct disorders, schizoaffective disorders, substance use disorder, and suicidal thoughts or behaviors. Specific disorders examined were ADHD, oppositional defiant, and other conduct disorders; anxiety disorders; and depression. We also identified and included a measure of suicidal thoughts and behaviors. Following the Agency for Healthcare Research and Quality's Chronic Conditions Warehouse guidance, we required one inpatient, one pharmacy, or two outpatient claims for each diagnosis. Definitions for these conditions are in Table S2.

Measures of health care use represent the health behaviors in Andersen's model. We included ED visits with a diagnosis for a mental health condition or suicidal thoughts and behaviors and outpatient utilization for school‐based encounters or mental health treatment. In Tennessee's Medicaid claims data, school‐based encounters are identified using a provider of service code, and outpatient mental health encounters are identified by adapting the algorithm used by Hugunin et al. to identify these visits in commercial claims data [30]. Definitions for these encounters are in Table S3. School‐based encounters do not have to be billed by an SBHC; they could represent community‐based providers seeing specific students in a school setting. Both types of encounters require parental consent. The difference between this scenario and an SBHC is that SBHCs provide comprehensive services to all students in the school. However, we hypothesize that SBHCs are associated with an increased probability of students having school‐based encounters. Both types of school‐based encounters require parental consent.

### 2.5 Statistical Analysis

To estimate the association of SBHC adoption with child mental health diagnoses and utilization, we used a DID design, which compares changes in outcomes in schools with an SBHC to changes in schools without an SBHC, before and after the SBHC's establishment. Methodological research has demonstrated that in the presence of staggered adoption of the intervention and heterogeneous treatment effects, standard DID models may produce biased effect estimates [31].

Given the staggered adoption of SBHCs in Tennessee and likely heterogeneous treatment effects, we employ a stacked DID design that accounts for both of these effects [32]. We included the two years before and five years after the implementation of an SBHC, which balances capturing the effects directly attributable to SBHC adoption with the possibility that it takes time to get these services in place and offered to all students in a district.

### 2.6 Sensitivity Analyses

We conduct two types of sensitivity analyses—one with respect to the design of the stacked DID estimator and another to the choice of the staggered adoption estimator. In the first, we exclude data from academic years (AY) 2019–2020 and 2020–2021, due to the disruptions of the COVID‐19 pandemic on school and healthcare. In the second, we use the Callaway & Sant'Anna estimator for staggered adoption DID situations [33]. Using this estimator, we also assess the validity of the parallel trends assumption, because the stacked DID estimator drops the period before adoption, leaving only one pre‐adoption time point, which allows for the assessment of differences in level but not trends.

The respective Institutional Review Boards approved this research, and we followed STROBE guidelines for observational studies. All analyses were conducted in Stata 18 (StataCorp LLC; College Station, TX).

## 3 Results

We identified 38 school districts with an SBHC from 2007 to 2019, representing 1296 (52.8%) of public schools in Tennessee. Three school districts, representing 109 (4.4%) schools, implemented an SBHC in 2006 or earlier and were excluded from our analysis. In general, districts that had an SBHC were larger than those that did not have an SBHC (Table 1). Specifics on districts, implementation year, and geographic location of SBHCs are in Table S4 and Figure S2.

**TABLE 1:** Districts and schools by SBHC status, 2006–2019.

	Number of districts	Number of schools in district	Percent of schools in TN
No SBHC by 2019	102	1008	41.1
SBHC adopted in each year:			
2007	0	0	0
2008	3	60	2.6
2009	4	650	28.1
2010	2	12	0.5
2011	1	95	4.1
2012	5	214	9.2
2013	1	9	0.4
2014	0	0	0
2015	2	91	3.9
2016	3	27	1.2
2017	3	27	1.2
2018	5	48	2.0
2019	9	95	4.1
TOTAL SBHC	38	1296	52.8

*Note:* Census information was collected by calling or emailing the director of coordinated school health in each school district in Tennessee. We asked the directors a standardized set of questions about SBHCs and when they were implemented. We received responses from 142 of the 147 (97%) school districts in Tennessee on the presence of an SBHC. We then calculated the number of districts and schools exposed to an SBHC during our study period, from 2006 to 2019. All schools in a district were considered ‘treated’ if the district had an SBHC. Schools with an SBHC prior to 2006 were excluded from our analyses.

### 3.1 Sample Characteristics

Schools in districts with an SBHC had a higher proportion of students identified in our data as Black (44.9 vs. 10.8%; *p* < 0.001) or Hispanic (4.7 vs. 3.2%; *p* < 0.001), economically disadvantaged (62.4 vs. 43.8%; *p* < 0.001), and with English as their second language (5.8 vs. 3.2%; *p* < 0.001) in 2007 (Table 2). Students in districts with an SBHC were less likely to identify as white (49.0 vs. 84.7%; *p* < 0.001) or be included in special education (15.0 vs. 19.5%; *p* < 0.001) compared to students in districts without an SBHC. A comparison of these characteristics between the linked sample and all students in Tennessee is available in Table S5. Linked students are more likely to be non‐white, economically disadvantaged, and in special education compared to all Tennessee public school students. However, they are less likely to be Hispanic, English language learners, and immigrants.

**TABLE 2:** School‐level demographic characteristics, health conditions, and health care use by SBHC status at baseline, 2007.

	Overall, % (SE)	No SBHC, % (SE)	SBHC, % (SE)	*p*
Demographic characteristics				
Female	48.4 (0.2)	48.4 (0.2)	48.3 (0.2)	0.92
Black	27.7 (0.8)	44.9 (1.3)	10.8 (0.5)	< 0.001
Hispanic	4.0 (0.1)	4.7 (0.3)	3.2 (0.2)	< 0.001
White	67.0 (0.8)	49.0 (1.3)	84.7 (0.6)	< 0.001
Other race	1.3 (0.1)	1.4 (0.1)	1.2 (0.1)	0.06
Inclusion in special education	17.3 (0.2)	15.0 (0.4)	19.5 (0.2)	< 0.001
English is not first language	4.5 (0.2)	5.8 (0.4)	3.2 (0.2)	< 0.001
Immigrant	0.6 (0.1)	1.0 (0.1)	0.2 (0.02)	< 0.001
Economically disadvantaged	53.4 (0.7)	43.6 (0.9)	63.4 (1.0)	< 0.001
Mental health (MH) diagnoses				
Any MH condition	12.0 (0.2)	12.5 (0.2)	11.5 (0.3)	0.002
ADHD	9.4 (0.1)	9.6 (0.2)	9.3 (0.2)	0.21
Anxiety	2.0 (0.05)	2.2 (0.06)	1.7 (0.08)	< 0.001
Depression	3.6 (0.1)	4.0 (0.1)	3.3 (0.1)	< 0.001
Suicidal thoughts/behaviors	0.05 (0.01)	0.04 (0.01)	0.06 (0.03)	0.40
Healthcare use				
ED visit: any MH condition	0.9 (0.3)	0.9 (0.05)	0.9 (0.04)	0.36
ED visit: suicidal thoughts/behaviors	0.2 (0.02)	0.2 (0.02)	0.2 (0.03)	0.37
Outpatient mental health visit	10.9 (0.2)	11.1 (0.2)	10.8 (0.3)	0.34
Telehealth	0.00 (0.00)	0.00 (0.00)	0.00 (0.00)	0.22
Psychotherapy	2.9 (0.07)	2.9 (1.1)	2.8 (0.09)	0.38
Medication use	11.4 (0.2)	12.3 (0.2)	10.5 (0.2)	< 0.001
School‐based encounter	1.2 (0.07)	0.9 (0.08)	1.4 (0.1)	< 0.001
*N* (schools)	1703	867	836	—

*Note:* Values represent the mean percentage of students in a school with these characteristics. Standard errors (SEs) are in parentheses. *P* values represent t‐tests of means for outcomes in 2007 between schools with an SBHC and schools without one. SBHC status was determined in 2021, the last year of our study period. Definitions for student characteristics, diagnoses, and healthcare use are in the Appendix materials. The Tennessee Department of Education changed the criteria to identify economically disadvantaged students during our study period. Before 2016 to 2017, they were identified according to their eligibility for free/reduced price lunch. Since 2017 to 2018, direct certification of students in economic disadvantage via family eligibility for federal public assistance programs requires having student social security numbers.

Abbreviations: ADHD, attention deficit and hyperactivity disorder; ED, emergency department; SBHC, school‐based health center; SE, standard error.

Overall, approximately 12% of students in our sample in 2007 (baseline year) were diagnosed with a mental health condition (Table 2), and this percentage is slightly higher in 2007 among districts that never adopt an SBHC (12.5 vs. 11.5%; *p* = 0.002). Students in districts without an SBHC had higher rates of all conditions examined compared to students in districts with an SBHC—ADHD (9.6 vs. 9.3%, respectively; *p* = 0.21); anxiety disorders (2.2 vs. 1.7%; *p* < 0.001); and depression (4.0 vs. 3.3%; *p* < 0.001). Only the rate of suicidal thoughts and behaviors (0.03 vs. 0.04%; *p* = 0.4) was lower in districts without an SBHC compared to those with one, but this difference is not statistically different from zero.

**TABLE 3:** Mechanisms suggested by DID estimates for the adoption of SBHCs on mental health outcomes.

Mechanism of SBHC				
Outcome	DID estimate (percentage points)	Primary prevention	Secondary prevention	Substitution
Diagnoses				
Any MH condition	−0.76**			
Anxiety	−0.47***			
ADHD	−0.50*			
Depression	−0.45**			
Suicidal thoughts/behaviors	−0.003			
Health care use				
ED visit for MH	−0.15*			
ED visit for suicidality	−0.01			
Outpatient MH visit	+0.95**			
Telehealth	−0.36***			
Psychotherapy	+0.31			
MH medication use	−0.69**			
School‐based visit	+0.91**			

*Note:* Shading represents the strength of the relationship between the proposed mechanism and the outcome, with darker colors being more suggestive of the mechanism at work than the lighter color. Difference‐in‐difference (DID) estimates are the weighted average treatment on the treated (ATT) from a stacked DID estimator [32]. Linear probability models were adjusted with school‐level sociodemographic characteristics, such as proportion of students identified as Black, low‐income, and for whom English is not their first language. Any mental health condition includes a diagnosis of ADHD, anxiety, bipolar disorder, depression, eating disorders, oppositional defiant and other conduct disorders, schizoaffective disorders, substance use disorder, and suicidal thoughts or behaviors. Outpatient mental health, telemental health, and psychotherapy visits were identified using an adapted version of the algorithm developed by Hugunin et al. (2022). Other utilization metrics were identified as those with a primary diagnosis of either a mental health or substance use disorder presenting in the ED. MH medication use is defined as filling any prescription for a medication used definitively to treat a mental health condition.

Abbreviations: ADHD, attention deficit and hyperactivity disorder; ED, emergency department; MH, mental health.

* *p* < 0.05.

** *p* < 0.01.

*** *p* < 0.001.

*Source:* Authors' analysis of Medicaid claims data linked to education records of school‐aged children in Tennessee from 2006 to 2022. Schools with an SBHC prior to 2006 were excluded from our analyses.

In general, the baseline rates of healthcare use were the same or higher in districts that did not adopt an SBHC relative to those that did (Table 2). Specifically, rates of ED use for mental health conditions (0.9%) and suicidal thoughts and behaviors (0.2%) were the same in districts with and without an SBHC, as were rates of psychotherapy (2.9%). Fewer students in districts with an SBHC had any outpatient mental health visit (10.8 vs. 11.1%; *p* = 0.34) or medication use for a mental health condition (10.5 vs. 12.3%; *p* < 0.001) compared to students in districts without an SBHC. Finally, students in districts with an SBHC were more likely to have a claim with the place of service code for a school compared to students in non‐SBHC districts (1.4 vs. 0.9%; *p* < 0.001) at baseline, which could indicate readiness and infrastructure to implement an SBHC. Every school in each year of the study period has at least one school‐based claim (Table S6).

### 3.2 DID Estimates

**FIGURE 1:**
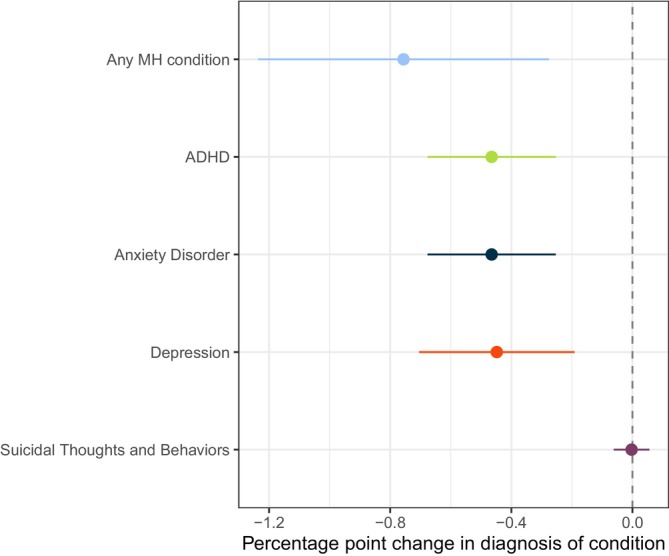
Changes in the prevalence of any mental health condition by SBHC status, 2006–2021. *Note:* Analysis conducted at the school‐academic year level. The dots represent the average treatment effect on the treated (ATT) from a DID analysis using a stacked DID estimator with 2 years of pre‐period data and 5 years of post‐period data. The ATT should be interpreted as the differential percentage point change in the diagnosis of these conditions in districts with an SBHC compared to those without an SBHC. The lines represent 95% confidence intervals; if these lines cross zero, the result is considered not statistically significant at the α = 0.05 level. Linear probability models were adjusted for district‐level sociodemographic characteristics, such as proportion of students identified as Black, low‐income, and for whom English is not their first language. Standard errors are calculated via wild cluster bootstrap at the district level. Any mental health condition includes a diagnosis of ADHD, anxiety, bipolar disorder, depression, eating disorders, oppositional defiant and other conduct disorders, substance use disorder, and suicidal thoughts or behaviors. Conditions were identified using medical (diagnosis & procedure codes) and pharmacy claims. We followed CMS' Chronic Conditions Warehouse guidance and required two outpatient, one inpatient, or one pharmacy claim with relevant codes in an academic year to classify a condition. *Source:* Authors' analysis of Medicaid claims data linked to education records of school‐aged children in Tennessee from 2006 to 2021. Schools with an SBHC prior to 2006 were excluded from our analyses. Abbreviations: ADHD, attention deficit and hyperactivity disorder; SBHC, school‐based health center.

In DID regression models, we found that the adoption of an SBHC was associated with a decrease in the probability of being diagnosed with any mental health condition by 0.76 percentage points (ppt; 95% CI: −1.24, −0.28) among students in a district with an SBHC relative to those in a district with no SBHC (Figure 1). This corresponded to a 6.6% relative decrease in the proportion of students diagnosed with any mental health condition. Examining the diagnosis of each mental health condition separately, we found that the implementation of an SBHC was associated with a decline of 0.47 ppt (95% CI: −0.68, −0.25) in the diagnosis of ADHD, which corresponds to a 5.4% relative reduction in the diagnosis of ADHD. A reduction in the probability of being diagnosed with anxiety disorder (−0.47 ppt; 95% CI: −0.26, −0.68) and depression (−0.45 ppt; 95% CI: −0.19, −0.71) was also associated with the adoption of an SBHC. These correspond with a 27.6% and 13.6% relative reduction from baseline, respectively. We found no statistically significant association between SBHC adoption and diagnoses for suicidal thoughts and behaviors (−0.003 ppt; 95% CI: −0.06,0.06). For all health outcomes, we found no violations of the parallel trends assumption using the Callaway & Sant'Anna estimator (Figure S4, Panels A–E).

**FIGURE 2:**
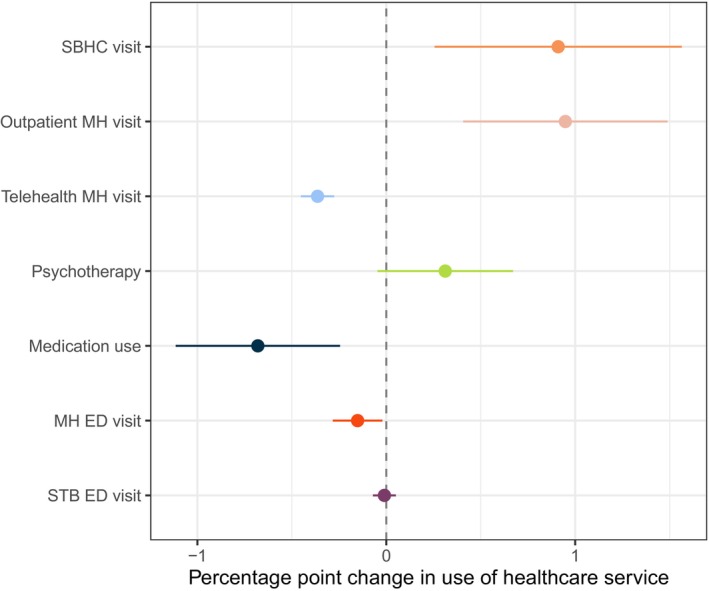
Change in healthcare use after an SBHC is adopted, 2006–2021. *Note:* Analysis conducted at the school‐academic year level. The dots represent the average treatment effect on the treated (ATT) from a DID analysis using a stacked DID estimator with 2 years of pre‐period data and 5 years of post‐period data. The ATT should be interpreted as the differential percentage point change in the diagnosis of these conditions in districts with an SBHC compared to those without an SBHC. The lines represent 95% confidence intervals; if these lines cross zero, the result is considered not statistically significant at the α = 0.05 level. Linear probability models were adjusted for district‐level sociodemographic characteristics, such as proportion of students identified as Black, low‐income, and for whom English is not their first language. Standard errors are calculated via wild cluster bootstrap at the district level. Outpatient mental health, telemental health, and psychotherapy visits were identified using an adapted version of the algorithm developed by Hugunin et al. (2022). Other utilization metrics were identified as those with a primary diagnosis of either a mental health or substance use disorder presenting in the ED. Medication use is defined as filling any prescription for a medication used definitively to treat a mental health condition. *Source:* Authors' analysis of Medicaid claims data linked to education records of school‐aged children in Tennessee from 2006–2021. Schools with an SBHC prior to 2006 were excluded from our analyses. Abbreviations: ADHD, attention deficit hyperactivity disorder; ED, emergency department; MH, mental health; SBHC, school‐based health center; STB, suicidal thoughts and behaviors.

The introduction of an SBHC was associated with changes in several types of healthcare use (Figure 2). First, the adoption of an SBHC was associated with increases in outpatient mental health and school‐based visits. Students in districts that adopted an SBHC were 0.91 ppt (95% CI: 0.25, 1.56) more likely to receive any school‐based healthcare visit. Similarly, SBHC adoption was associated with a 0.95 ppt (95% CI: 0.41,1.49) increase in the probability of a student receiving any outpatient visit for mental health compared to students in districts without an SBHC; this is an 8.8% relative increase in the percent of students who have such a visit. SBHC adoption was also associated with an increase in the probability a student received psychotherapy (0.31 ppt; 95% CI: −0.5 0.67) in districts with an SBHC compared to districts without; this difference is not statistically different from zero, however.

Conversely, the adoption of an SBHC was associated with a differential decrease in the probability of students receiving telemental health visits of 0.36 ppt (95% CI: −0.28, −0.45) in districts that adopted an SBHC vs. districts that did not, which represents a 120% relative decline. Reductions in the use of medications for mental health likewise declined; after the adoption of an SBHC, the percent of students who had a prescription for a mental health condition declined by 0.69 ppt (95% CI: −0.24, −1.12), a relative reduction of 6.6%.

Lastly, the adoption of an SBHC was associated with differential declines in the probability of having a mental health ED visit (0.15 ppt; 95% CI: −0.02, −0.28), which corresponds to a 25% relative decrease in the likelihood of a mental health‐related ED visit. Conversely, SBHC adoption was not associated with changes in the probability of an ED visit for suicidal thoughts and behaviors (0.01 ppt; 95% CI: −0.07, 0.05). Like the health outcomes, we found no evidence of violation of the parallel pre‐trends assumption among any of the healthcare use outcomes, apart from psychotherapy (Figure S5, Panels A–G). However, the results for psychotherapy are null, meaning that our results are likely biased toward the null.

### 3.3 Sensitivity Analysis Results

Overall, our results are robust to restricting to the pre‐pandemic period (AY 2006–2019; Table S7). We estimated statistically significant associated decreases in the percentage of students with any mental health diagnosis (−0.96 vs. −0.76 for analyses ending in AY2019 and AY2021, respectively) and ADHD (−1.0 vs. −0.50 for analyses ending in AY2019 and AY2021, respectively) in both analyses. However, when we restricted to the pre‐pandemic period, our estimates for the diagnosis of anxiety (−0.22 vs. −0.47) and depression (−0.18 vs. −0.45) were roughly half the size of the full study period (AY2006‐2021) and no longer statistically significant.

When examining the utilization data, we found that SBHC adoption was, unsurprisingly, associated with increases in school‐based health visits nearly twice as large in the pre‐pandemic period compared to the full study period (1.64 vs. 0.95 ppt). Similarly, we found effect sizes for mental health‐related ED visits twice the size in the pre‐pandemic period relative to the full study period (−0.33 vs. −0.15 ppt). This was likely due to the lack of access to school‐based health services combined with the uncertainty and anxiety induced by the pandemic. Thus, our estimates of SBHC adoption on mental health‐related ED visits were likely conservative. With respect to outpatient mental health visits, estimates that included AY2020 and AY2021 were nearly twice the size of estimates (0.95 vs. 0.44 ppt) that did not include the pandemic period. Additionally, the estimates that include the pandemic period are statistically significant while those from the sensitivity analyses are not. Again, these differences are likely due to the pandemic's disruption on outpatient health services.

We selected the stacked DID as our primary estimation method because it allowed us to specify the same number of pre‐treatment and post‐treatment periods (two pre and five post) for each district, whereas the Callaway & Sant'Anna method includes all available post‐period years in its estimation. Therefore, some districts that adopted an SBHC early in the study period have twice as many post‐adoption years (10 vs. 5) using the Callaway & Sant'Anna estimator compared to the stacked DID estimator. This large number of post‐adoption years yielded noisy estimates in the later post‐period years that are likely driven by the sample composition of schools in these years rather than the latent effects of SBHCs. The estimated treatment effects were consistent across the stacked DID and Callaway & Sant'Anna methods for our outcomes related to the diagnosis of any mental health condition and ADHD, although not for healthcare utilization. This was not unexpected given the differences between these methods in the time periods included in estimation.

## 4 Discussion

In this study, we found that the adoption of SBHCs was associated with a decline in mental health diagnoses in districts with these resources in Tennessee. Specifically, we estimated a 6.6% relative reduction in the diagnosis of any mental health conditions associated with districts that implemented an SBHC compared to districts that did not. We found consistent declines across most of the individual mental health conditions (e.g., ADHD, anxiety, depression) we examined, with suicidality as the exception. These findings were consistent with previous literature on SBHCs, which has demonstrated that SBHCs are effective in increasing access to preventive health services and helping students manage conditions amenable to types of services SBHCs provide, including mental health conditions [11, 13, 18, 19, 20, 34, 35, 36]. SBHCs could affect the prevalence of mental health outcomes in two ways, which have different policy and practice implications (Table 3).

First, SBHCs could provide students with tools to identify, address, and manage their health stresses, which could prevent these stresses from reaching a clinical or diagnosable threshold.

Interviews in our study with directors of coordinated school health (CSH) in Tennessee schools indicated that they have used SBHC funding to increase mental health staffing—such as social workers, therapists, and behavioral specialists—to enable more students to interact with professionals who can address their needs and prevent conditions from reaching a diagnosable level [37]. CSH staff frequently described efforts to deliver regular preventive programming to equip students with tools to understand and manage their mental health, including on topics such as suicide awareness and prevention and bullying prevention.

Second, SBHCs could be acting as substitutes for and/or complements to community‐based health care services. If SBHCs are not billing Medicaid at the same level as community‐based providers, diagnoses of mental health conditions may decline in administrative claims. This does not necessarily imply that diagnoses are going down or that cases of mental health conditions are being diverted. Rather, they are being recorded with less frequency. Indeed, several SBHCs have highlighted in our interviews that the administrative burden of billing Medicaid is substantial and acts as a barrier for many SBHCs to accept or bill insurance [37]. They have instead looked for funding sources that allow them to extend their services to students regardless of their insurance status. We, however, find that SBHC adoption is associated with increases in school‐based visits.

Our study, one of the first to look at SBHCs' impact on healthcare use, primarily supports the first mechanism—that SBHCs are effective at increasing students' ability to understand and manage their mental health stresses. First, we estimated a 25.0% relative reduction in the proportion of students with an ED visit for a mental health condition, as well as a 9.4% relative decrease in the proportion of students receiving a mental health prescription after the adoption of an SBHC. Similarly, we estimated a 12% relative increase in the proportion of students who had an outpatient mental health visit in the community after the adoption of an SBHC. We found no changes in the incidence of ED visits for suicidal thoughts/behaviors, which is a rarer outcome. Thus, we may be underpowered to detect an effect. Taken together, these suggest that SBHCs may be effective as both primary and secondary prevention mechanisms, reducing the clinical manifestation of mental health conditions and identifying and referring students with greater need to outpatient mental health services in the community (where diagnoses would be recorded in claims).

Other utilization results, however, suggest that SBHCs may be acting as a substitute for community‐based providers who would bill Medicaid. In addition to reductions in ED visits and medication use, we also estimated a reduction in the proportion of students who received a telemental health visit after the adoption of an SBHC. From our interviews with CSH staff, we heard that telehealth played an increasingly large role in providing mental health services to students in schools even before the COVID‐19 pandemic, which may be replacing telemental health services provided through community‐based Medicaid providers [37].

A comparison of our sensitivity analyses, which restrict to the pre‐pandemic period, provides additional evidence for this hypothesis. The increases in the outpatient mental health visits are twice as large in the full study period, compared to the pre‐pandemic period. However, this could also be driven by increased mental health needs and heightened awareness about these needs among caregivers during the pandemic rather than by substitution away from unbilled, school‐based visits during the pandemic. Still, most of the evidence from our analyses suggests that SBHCs play an important role in the prevention, identification, and referral to treatment for mental health rather than a simple substitution away from Medicaid‐billed services.

These results provide important directions for additional research and policy. First, additional research on which students are reached and best served via SBHCs is critical. If changes in outcomes and utilization are concentrated among underserved students, then additional financial and personnel resources could be dedicated to SBHCs to narrow existing differences in mental health treatment. Prior research has found that students who are from low‐income households, identify as a racial/ethnic minority, or are publicly insured (i.e., have Medicaid or CHIP) are more likely to receive care exclusively from an SBHC [7]. More than a third of SBHCs are located in rural areas, which are unlikely to have adequate community‐based mental health services and could potentially benefit the most from investment in mental health services in SBHCs [38].

Second, future research on the effects of SBHCs on child mental health outcomes should examine in greater depth the resources necessary to achieve the positive mental health outcomes we observe. Typically, SBHCs are categorized in one of two ways: on‐site, fixed facility school‐based centers and off‐site or school‐linked centers, including mobile health and telehealth [39]. While SBHCs have provided primary care services since their inception, the percentage of SBHCs providing mental health services has increased by nearly 50 percentage points since 2010 [40]. Understanding the modalities, kinds of services, staff, and resources that SBHCs need to provide to fully support student mental health is critical to addressing the existing mental health crisis, particularly in a policy environment operating in a scarcity mindset where fewer resources are likely available to schools and state Medicaid agencies.

Lastly, it is also important to note that many school districts in Tennessee that do not have an SBHC were similarly striving to increase their mental health staffing in schools over our study period, drawing on resources provided through the statewide CSH program, the Tennessee Department of Mental Health and Substance Abuse Services' School‐Based Behavioral Health Liaison program, and other time‐limited grant opportunities. Therefore, the contrast in service capacities between districts with and without SBHCs may be limited in some cases. Given that we still find effects of SBHCs, even marginal increases in these capacities may be beneficial for student wellbeing.

### 4.1 Limitations

Like all observational research, our study has limitations. First, this is a school‐level, intent‐to‐treat analysis. We do not have information on which students within a district receive services, nor do we directly observe the kinds of services students receive from an SBHC. This may result in an underestimate of the effect of SBHCs on mental health diagnoses. Second, there may be unobserved sources of selection bias. School districts that implemented an SBHC may have differed in ways we could not observe with available data that led them to implement an SBHC. Specifically, we did not have data on whether a student was eligible for Medicaid due to household income, disability, or foster care placement. Third, our analysis relies on untestable causal assumptions. Finally, linked students are demographically different from all students in TN schools; thus, our results may not generalize to districts with larger populations of Hispanic children, English language learners, and immigrants.

## 5 Conclusion

SBHCs may be one mechanism through which the youth mental health crisis in the U.S. (and abroad) can be addressed. Children and adolescents spend more waking time in school than at home, providing an opportunity to meet the mental health needs of all school‐aged students. Timely and adequate resources are needed to bolster the infrastructure of SBHCs and ensure they can sustain their services in this critical time of need, particularly given funding [25] and behavioral health staffing shortages [40]. Tennessee ranks near the bottom of the states in youth mental healthcare access, with more than 70% of youth with mental health needs going untreated, underscoring the urgency of additional investments [40]. Recent cancellations of school‐based mental health grants [41], the restructuring of the Department of Education [42], and the signing of H.R. 1 may limit federal funding for school‐based health care services [41, 42, 43]. Specifically, H.R. 1 limits the use of provider taxes as a source of state Medicaid spending in expansion states, which will put tens of billions in federal Medicaid funding at risk in fiscal year 2026 [44]. Since states must maintain balanced budgets, federal funding decreases will require states to raise revenue, cut funding in other areas, or limit Medicaid benefits/eligibility. How and where states will reduce spending is unclear, but states will have difficult decisions to make regarding state education and Medicaid policy, and programs and services that address the child mental health crisis in the United States will be vulnerable. To meet the policy challenge head on, we must identify ways to improve the mental health and wellbeing of all American children—our research suggests that school‐based health may be one way to do so.

## Conflicts of Interest

The authors declare no conflicts of interest.

## Supporting information


**Data S1:** hesr70042‐sup‐0001‐Supinfo1.docx.

## Data Availability

The data that support the findings of this study are available from Tennessee Department of Education, Tennessee Department of Health, and Tennessee's Medicaid program, TennCare. Restrictions apply to the availability of these data, which were used under license for this study. Data are available from the author(s) with the permission of Tennessee Department of Education, Tennessee Department of Health, and Tennessee's Medicaid program, TennCare.
