# Supplementary material for: Association of School‐Based Health Center Availability With Child Mental Health Outcomes
Source: Health Serv Res. 2025 Sep 11;61(1):e70042. doi: 10.1111/1475-6773.70042 (PMC12588070; doi:10.1111/1475-6773.70042)
Supplement: Supplementary file 1 — Data S1: hesr70042‐sup‐0001‐Supinfo1.docx. [file HESR-61-e70042-s001.docx]

**Supplemental Materials for:**

*The association of school-based health centers with child mental health and healthcare utilization outcomes*

August 26, 2025

**eFigure 1.** Application of the Andersen Behavioral Model of Health Care Use to adoption of school-based health centers (SBHCs) in TN

**PREDISPOSING
CHARACTERISTICS**

**USE OF HEALTH SERVICES**

**NEED**

**ENABLING RESOURCES**

DEMOGRAPHIC
Sex
Race/Ethnicity
English language learner
Age
Special education status

PERSONAL/FAMILY
Economic disadvantage
Housing status
Immigration status

School-based health services
Outpatient MH care
Telemental health care
Psychotherapy
Medication treatment
Emergency department

PERCEIVED

COMMUNITY
Presence of an SBHC
Coordinated School Health
Other healthcare providers
TN policies on billing
TN policies on consent

EVALUATED
Diagnosis of any mental health condition

Diagnosis of ADHD, anxiety, depression, suicidality

**eTable 1.** Operationalization of predisposing factors

| **Predisposing characteristics** | **Source** | **Coding** |
| --- | --- | --- |
| Sex | Administrative education records, self-reported by parent /guardian upon school enrollment | Binary, 1 = female |
| Non-Hispanic white |  | Binary, 1 = NH white |
| Non-Hispanic Black |  | Binary, 1 = NH Black |
| Some other race |  | Binary, 1 = some other race |
| Hispanic/Latino |  | Binary, 1 = Hispanic |
| Inclusion in special education | Administrative education records, indicating the presence of an IEP for 1 of the 16 disability categories | Binary, 1 = included in special education |
| English as a second language | Administrative education records | Binary, 1 = English language learner |
| Immigrant | Administrative education records | Binary, 1 = immigrant |

**eTable 2**. Definitions of mental health outcomes

| **Health Measure** | ***ICD-9-CM* Codes** | ***ICD-10-CM* Codes** | **Procedure Codes** | **Prescription Drugs** |
| --- | --- | --- | --- | --- |
| **Attention deficit hyperactivity disorder [ADHD] and conduct disorders** | 312.0-312.9; 313.81; 314.0-314.9 | F63.0-F63.9; F90.0-F90.9; F91.0-F91.9 | N/A | Amphetamine; Atomoxetine;  Dexmethylphenidate hydrochloride; Dextroamphetamine sulfate; Dextroamphetamine-amphetamine; Lisdexamfetamine dimesylate; Methylphenidate hydrochloride; Guanfacine hydrochloride |
| **Bipolar**  **disorder** | 296.00-296.06; 296.10-296.16; 296.40-296.7;  296.80-296.99 | F30.1-F30.9; F31.0-F31.9; F33.8; F34.8; F34.89;  F34.9; F39.0 | N/A | Lithium; Divalproex sodium; Lamotrigine |
| **Anxiety disorders** | 293.84; 300.00-300.09; 300.1;  300.2-300.29; 300.3; 300.5;  300.89; 300.9; 308-308.9; 309.81; 313-313.83 | F4310-F4312; F06.4; F40.0-F40.9; F41.0; F41.1-F41.9; F42.0-F42.9; F43.0,F43.12; F44.9; F45.8; F48.8; F48.9; F93.8; F99; R45.2;  R45.5; R45.6; R45.7 | N/A | Clomipramine hydrochloride; Duloxetine hydrochloride; Fluoxetine hydrochloride; Fluoxetine-olanzapine; Fluvoxamine; Imipramine; Lorazepam; Paroxetine hydrochloride; Sertraline hydrochloride; Venlafaxine |
| **Depression** | 296.2-296.36; 296.51-296.56; 296.60-296.66; 296.89; 298.0; 300.4; 309.1;  311 | F31.30-F31.81; F32.0-F32.9;  F33.0-F33.9; F34.1; F43.21-F43.23 | N/A | desvenlafaxine succinate; levomilnacipran; nefazodone phenelzine sulfate; tranylcypromine sulfate; vilazodone hydrochloride; Vortioxetine; hydrochloride; Amoxapine; Bupropion hydrochloride; Citalopram; Clomipramine hydrochloride; Desipramine; Doxepin hydrochloride; Duloxetine hcl; Escitalopram; Fluoxetine hydrochloride; Fluoxetine-olanzapine; Fluvoxamine; Imipramine; Maprotiline hydrochloride; Milnacipran; Mirtazapine; Nortriptyline hydrochloride; Paroxetine hydrochloride; Protriptyline hydrochloride; Selegiline hydrochloride; Sertraline hydrochloride; Trazodone hydrochloride; Trimipramine maleate; venlafaxine |
| **Suicidal thoughts and behaviors** | E950-E95.29; E95.30-E95.89;  E95.9; V6284 | R45.851; T14.91-T14.91XS  T36.0X2A-T50.Z92S; T51.0X2A-T65.92XS; T71.112A-T71.232S; X71.0XXA-X71.9XXS;  X72XXXA-X74.9XXS; X75.XXXA-X77.9XXS; X78.0XXA-X78.9XXS; X79.XXXA-X79.XXXS; X80.XXXA-X81.8XX5;  X82.8XXA-X83.0XXS; X83.1XXA-X83.1XXS; X83.2XXA-X83.8XXS | N/A | N/A |
| **Substance Use Disorders** | 292.0; 292.11-292.9;  304.00-304.92; 305.20-305.92; 648.30-648.34; 665.50-655.53;  760.72, 760.73, 760.75;  779.5; 965.00-965.02, 965.09;  E85.00-E85.02; E85.41;  E93.50, E93.51; V65.42 | F11.10-F11.99; F12.10-F12.99;  F13.10-F13.99; F14.10-F14.99;  F15.10-F15.99; F16.10-F16.99;  F18.10-F18.99; F19.10-F19.99; F55.0-F55.8; O35.5XX0-O35.5XX9; O99.320-O99.325; P04.4, P04.49; P96.1-P96.2; T40.0X1A-T40.991A;  Z71.41-Z71.52 | ICD9-PCS: 946,  9464,  9465,  9466,  9467,  9468,  9469    ICD-10-PCS: HZ2ZZZZ HZ30ZZZ, HZ31ZZZ, HZ32ZZZ, HZ33ZZZ, HZ34ZZZ, HZ35ZZZ, HZ36ZZZ, HZ37ZZZ, HZ38ZZZ, HZ39ZZZ, HZ3BZZZ, HZ40ZZZ; HZ93ZZZ, HZ96ZZZ | Naloxone; Disulfiram; Naltrexone; Buprenorphine; Acamprosate; |

**eTable 3.** Definitions of healthcare utilization outcomes

| **Description** | **Claim Requirements** | **Place of Service Codes** | **Provider type/specialty codes** | **Procedure Codes** |  |
| --- | --- | --- | --- | --- | --- |
| **EMERGENCY DEPARTMENT** | | | |  |  |
| Mental health ED visit | Outpatient ED claim with mental health diagnosis attached | N/A | N/A | N/A |  |
| Substance use ED visit | Outpatient ED claim with substance use diagnosis attached | N/A | N/A | N/A |  |
| ED visit for suicidal thoughts/ behaviors | Outpatient ED claim with suicidal thoughts/behaviors diagnosis attached | N/A | N/A | N/A |  |
| **INPATIENT** | | | | | |
| Mental health inpatient stay | Inpatient claim with mental health diagnosis attached | N/A | N/A | N/A |  |
| Substance use inpatient stay | Inpatient claim with substance use diagnosis attached | N/A | N/A | N/A |  |
| Inpatient stay for suicidal thoughts / behaviors | Inpatient claim with suicidal thoughts/behaviors diagnosis attached | N/A | N/A | N/A |  |
| **OUTPATIENT** | | | | | |
| School-based encounter | Outpatient, non-ED claim with place of service (POS) code for schools | N/A | N/A | N/A |  |
| Mental health outpatient visit | Must meet one the following two conditions:   1. Criteria 1 for place of service AND provider specialty OR type 2. Criteria 2 for place of services | Criteria 1 – Place of service:  POS: Telehealth, School, Office, Home, Mobile Unit, Off Campus-Outpatient Hospital, On Campus-Outpatient Hospital, Independent Clinic, Federally Qualified Heath Care  Criteria 2 – Place of service: Psychiatric Facility Partial Hospitalization, Psychiatric Facility Partial Hospitalization | Criteria 1 – Provider specialty: Psychiatric; Mental Health Inpatient Hospital; Behavioral Health Organization; Outpatient Mental Health Clinic; Community Mental Health Center; Psychologist; Clinical Psychologist; Health Service Provider in Psychology; Certified Clinical Social Worker; Certified Social Worker; Psychiatric Nurse; Marriage and Family Counselor; School Corporation; Licensed Practical Counselor; Behavioral Analyst; Psychologist-Cognitive & Behavioral; Mental Health Case Management; Psychiatrist; Psychiatry; Licensed Psychiatric Technician  Criteria 1 - Provider type: Mental Health Provider, Therapist - Non-Certified, Counselor | N/A |  |
| Psychotherapy | Must meet the following conditions:   1. One of the following provider specialty *or* provider type codes 2. One of the following place of service codes 3. One of the following CPT codes | Telehealth, Office, Home, Mobile Unit, Off Campus-Outpatient Hospital, On Campus-Outpatient Hospital, Independent Clinic, Federally Qualified Heath Care, Community Mental Health Center | Provider Specialty: Psychiatry; Mental Health Inpatient Hospitals; Behavioral Health Organization; Outpatient Mental Health Clinic; Community Mental Health Center; Psychology; Clinical Psychology; Health Service Provider in Psychology; Certified Clinical Social Worker; Certified Social Worker; Psychiatric Nurse; Marriage and Family Counselor; School Corporation; Licensed Practical Counselor; Behavioral Analyst; Psychologist-Cognitive & Behavioral; MH Case Mgmt; Licensed Psychiatric Technician  Provider Type: Mental Health Provider, Therapist - Non-Certified, Counselor | 90832-90834, 90836-90840, 90846, 90847, 90849, 90853, 90887 |  |
| Telehealth for mental health | Must meet the following conditions:   1. One of the following provider specialty *or* provider type codes 2. One of the following place of service codes 3. One of the following CPT codes | Place of service: Telehealth services provided thru telecom system; Telehealth provided in patient's home | Provider Specialty: Psychiatry; Mental Health Inpatient Hospitals; Behavioral Health Organization; Outpatient Mental Health Clinic; Community Mental Health Center; Psychology; Clinical Psychology; Health Service Provider in Psychology; Certified Clinical Social Worker; Certified Social Worker; Psychiatric Nurse; Marriage and Family Counselor; School Corporation; Licensed Practical Counselor; Behavioral Analyst; Psychologist-Cognitive & Behavioral; Mental Health Case Management; Licensed Psychiatric Technician  Provider type: Mental Health Provider, Therapist - Non-Certified, Counselor | 99201-99205, 99211-99215, 99241-99245 90791-90792, 90832-90834, 90836-90840, 90846, 90847, 90849, 90853, 90887  With modifiers used in telehealth billing in Tennessee. |  |
| Mental health medication use | Pharmacy claim for medication used to identify mental health conditions |  | N/A | N/A |  |

**eTable 4.** School districts in TN with SBHCs and year of implementation

| **Year** | **Districts adopting an SBHC** | **Included in analysis as treated?** |
| --- | --- | --- |
| 1995 | Hancock County | No – not included in analysis at all |
| 2001 | Monroe County | No – not included in analysis at all |
| 2006 | Warren County | No – not included in analysis at all |
| 2008 | Bradley County, Sevier County | Yes |
| 2009 | Madison County, Memphis-Shelby County | Yes |
| 2010 | Gibson County, Trenton Special School District (Gibson County) | Yes |
| 2011 | Knox County | Yes |
| 2012 | Bells City School District (Crockett County), Crockett County, Haywood County, Unicoi County, Metro Nashville Public Schools | Yes |
| 2013 | Cleveland City Schools (Bradley County) | Yes |
| 2014 | Lauderdale County (AWARE grant implemented) | Yes |
| 2015 | Dyersburg City Schools (Dyer County), Hamilton County | Yes |
| 2016 | Humboldt City Schools (Gibson County), Obion County, Tipton County | Yes |
| 2017 | Bradford Special School District (Gibson County), Greene County, Tullahoma City Schools (Coffee/Franklin County) | Yes |
| 2018 | Bristol City Schools (Sullivan County), Dickson County, Dyer County, Franklin County, Lauderdale County, Manchester City Schools (Coffee County) | Yes |
| 2019 | Hawkins County, Jefferson County, Newport City Schools (Cocke County), Putnam County, Sequatchie County | Yes |
| 2020 | Carter County, Decatur County, Grainger County, Greeneville City Schools (Greene County), Hardeman County, Lake County, Maury County, Milan City Schools (Gibson County), Murfreesboro City Schools (Rutherford County), Rhea County, | No – included in comparison group |
| 2021 | Fayette County, Hardin County, Henry County, Jackson County, Oneida City Schools (Scott County), Rogersville City Schools (Hawkins County), Rutherford County, West Carroll Special School District (Carroll County) | No – included in comparison group |
| 2022 | Benton County, Cannon County, Elizabethton City Schools (Carter County), Fentress County, Lenoir City Schools (Loudon County), Morgan County, Roane County | No – included in comparison group |
| 2023 | Claiborne County | No – included in comparison group |
| — | Alamo City School (Crockett County), Anderson County, Arlington Community Schools (Shelby County), Athens City Schools (McMinn County), Bartlett City Schools (Shelby County), Bedford County, Bledsoe County, Blount County, Campbell County, Carroll County, Cheatham County, Clarksville-Montgomery County, Clay County, Clinton City Schools (Anderson County), Cocke County, Coffee County, Collierville City Schools (Shelby County), Cumberland County, DeKalb County, Etowah City Schools (McMinn County), Fayetteville City Schools (Lincoln County), Franklin Special School District (Williamson County), Giles County, Grundy County, Hamblen County, Henderson County, Hickman County, Hollow Rock-Bruceton City Schools (Carroll County), Houston County, Huntingdon Special School District (Carroll County), Johnson City Schools (Washington County), Johnson County, Kingsport City Schools (Sullivan/Hawkins County), Lakeland City Schools (Shelby County), Lawrence County, Lebanon Special School District (Wilson County), Lewis County, Lexington City Schools (Henderson County), Lincoln County, Macon County, Loudon County, Marshall County, Maryville City Schools (Blount County), McKenzie City Schools (Carroll County), McMinn County, McNairy County, Meigs County, Millington Municipal Schools (Shelby), Moore County, Oak Ridge City Schools (Anderson/Roane County), Overton County, Paris City Schools (Henry County), Perry County, Pickett County, Polk County, Richard City Schools (Marion County), Robertson County, Scott County, Smith County, Carroll County, Stewart County, Sullivan County, Sumner County, Sweetwater City Schools (Bradley County), Trousdale County, Union City Schools (Obion County), Union County, Van Buren County, Washington County, Wayne County, White County, Wilson County, Williamson County | No – included as comparison group |

**eFigure 2.** Map of SBHCs and AWARE grants in Tennessee


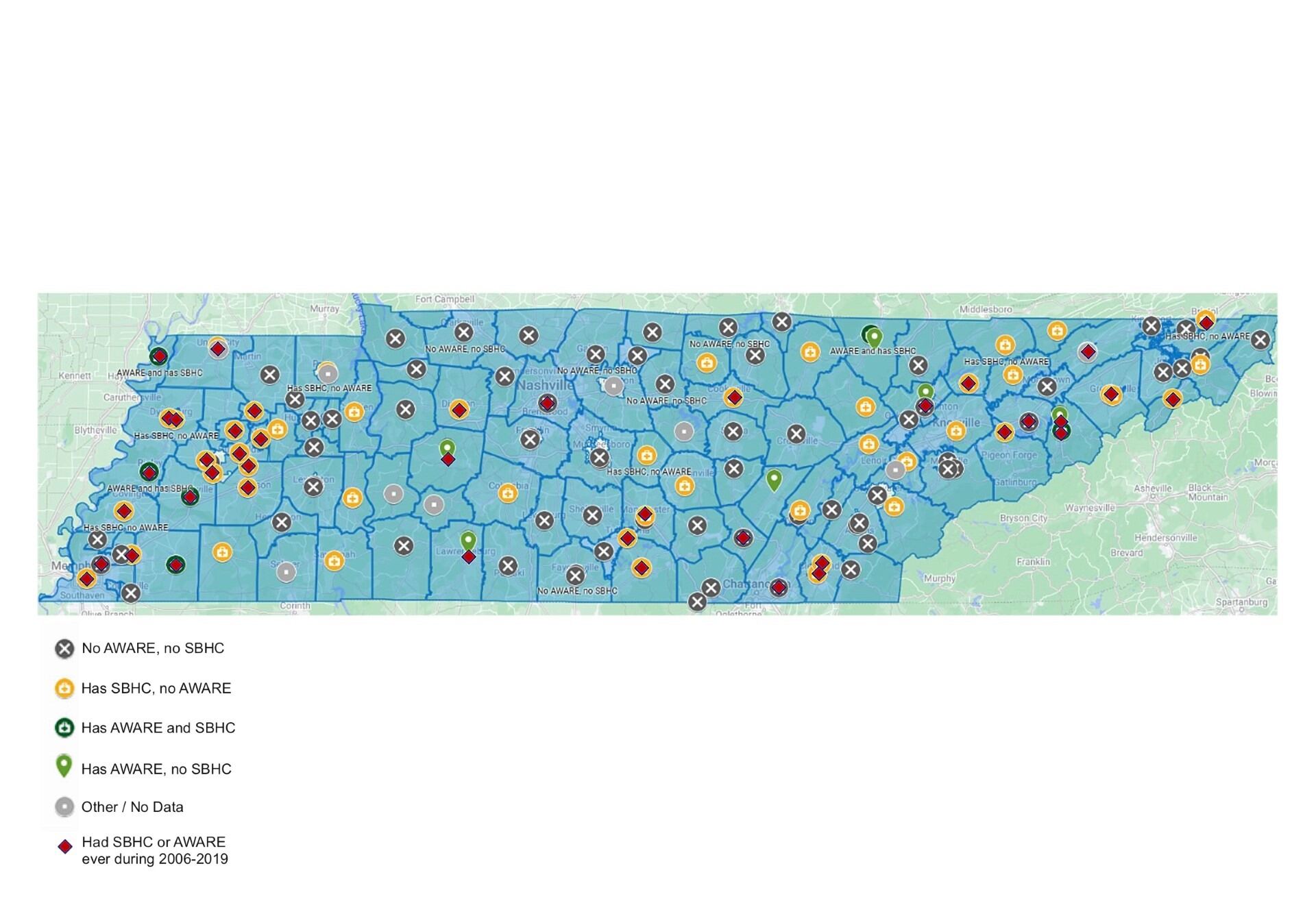


In treated group if SBHC adopted between 2008-2019

In comparison group

AWARE grantees in Wave 1 are excluded from analysis

In treated group if SBHC adopted between 2008-2019

In comparison group

In treated group

**eTable 5.** Comparison of student sample between linked students and all students

|  | **Overall** | |  | **SBHC** | | **No SBHC** | |
| --- | --- | --- | --- | --- | --- | --- | --- |
| **Characteristics (2007)** | *Linked  (%)* | *All  (%)* | *p-value* | *Linked (%)* | *All (%)* | *Linked  (%)* | *All (%)* |
| Female | 50.0 | 48.5 | p<0.001 | 49.9 | 48.6 | 50.1 | 48.4 |
| Black | 32.8 | 25.9 | p<0.001 | 50.6 | 42.0 | 11.6 | 9.2 |
| Hispanic | 4.2 | 4.8 | p<0.001 | 4.7 | 5.5 | 3.6 | 4.1 |
| White | 61.8 | 67.2 | p<0.001 | 43.3 | 50.3 | 83.7 | 84.8 |
| Other race | 1.3 | 2.1 | p<0.001 | 1.3 | 2.2 | 1.2 | 1.9 |
| Economically-disadvantaged | 54.0 | 40.3 | p<0.001 | 62.3 | 49.3 | 44.2 | 30.9 |
| Special Education | 16.5 | 12.9 | p<0.001 | 14.4 | 11.6 | 18.9 | 14.3 |
| English language learner | 4.8 | 7.7 | p<0.001 | 6.0 | 9.3 | 3.3 | 6.1 |
| Immigrant | 0.8 | 1.4 | p<0.001 | 1.2 | 2.1 | 0.2 | 0.7 |

SOURCE/NOTES **Source:** Authors’ analysis of education records for public school students in TN and a subset of those linked to Medicaid claims data. **Notes:** p-values are from a two-tailed t-test of means. Linked students are those for whom we can link/match Medicaid enrollment records to school enrollment records. All students are those identified in school records only.

**eTable 6.** Comparison of school-based claims by SBHC status, each year and overall, 2006-2021

| **Year** | **School-based Claims among**  **Schools with SBHC** | | | | **School-based Claims among**  **Schools without SBHC** | | | |
| --- | --- | --- | --- | --- | --- | --- | --- | --- |
|  | *Mean* | *Median* | *Min* | *Max* | *Mean* | *Median* | *Min* | *Max* |
| **2006** | 405.6 | 400 | 38 | 1368 | 404.8 | 360 | 1 | 2127 |
| **2007** | 402.7 | 382.5 | 41 | 1385 | 407.9 | 358 | 1 | 2193 |
| **2008** | 411.3 | 366 | 11 | 1394 | 411.2 | 366 | 1 | 2265 |
| **2009** | 472.52 | 420 | 5 | 1805 | 387.4 | 352 | 1 | 2157 |
| **2010** | 462.3 | 399 | 1 | 1816 | 388.5 | 350.5 | 2 | 1819 |
| **2011** | 450.2 | 408 | 5 | 1804 | 384.4 | 345 | 1 | 1901 |
| **2012** | 439.7 | 395 | 2 | 1731 | 373.2 | 338.5 | 1 | 1580 |
| **2013** | 438.5 | 389.5 | 7 | 2438 | 374.2 | 339 | 1 | 1566 |
| **2014** | 427.1 | 383 | 1 | 2069 | 374.08 | 338 | 2 | 1538 |
| **2015** | 430.8 | 387 | 1 | 1622 | 375.2 | 336 | 5 | 1864 |
| **2016** | 432.8 | 393.5 | 1 | 2216 | 370.1 | 326.5 | 2 | 1901 |
| **2017** | 428.9 | 383 | 5 | 2194 | 363.3 | 321 | 1 | 1608 |
| **2018** | 418.7 | 366 | 1 | 2051 | 363.9 | 323 | 2 | 1550 |
| **2019** | 411.1 | 359.5 | 1 | 2050 | 353.7 | 313 | 2 | 1500 |
| **2020** | 401.7 | 356 | 1 | 2070 | 349.7 | 307 | 1 | 1495 |
| **2021** | 402.5 | 353.5 | 4 | 1851 | 336.4 | 292 | 2 | 2049 |
| **2006-2021** | 426.0 | 376 | 1 | 2438 | 361.8 | 325 | 1 | 1686 |

SOURCE/NOTES **Source**: Authors’ analysis of Medicaid claims data linked to administrative education data in Tennessee. **Notes** School-based claims were those with a place of service code indicating a school (POS = 3 in Tennessee Medicaid claims data). Data were linked using a probabilistic algorithm that captures approximately 70% of all public-school students in Tennessee.
Abbreviations: SBHC = school-based health center

**eFigure 4.** Event studies for mental health diagnoses

Panel A. Any mental health condition


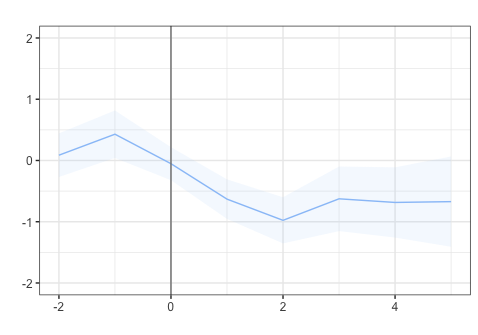


Percentage point difference
 in diagnoses

Panel B. Anxiety


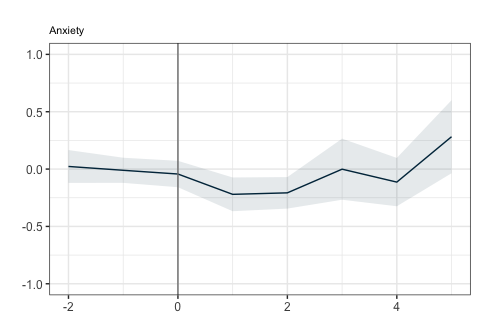


Percentage point difference
 in diagnoses

Year relative to SBHC adoption

Panel C. Depression


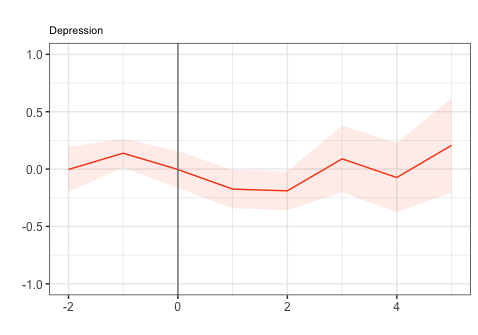


Percentage point difference
 in diagnoses

Panel D. ADHD
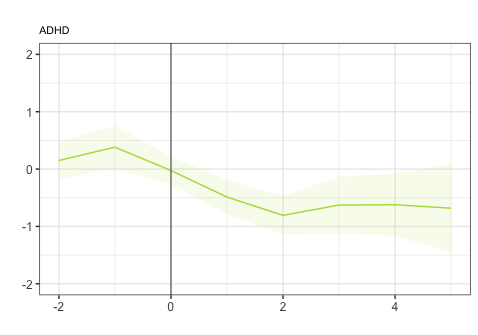


Year relative to SBHC adoption

Percentage point difference
 in diagnoses

Panel E. Suicidal thoughts and behaviors

**
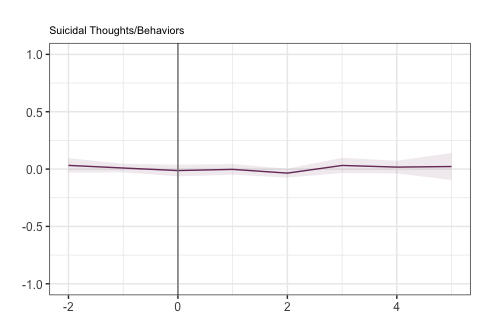
**

Percentage point difference
 in diagnoses

SOURCE/NOTES **Source** Authors’ analyses of linked Medicaid claims and administrative education data in Tennessee between 2006-2021. **Notes** Lines represent the differential estimates between from the districts that adopt and SBHC and those that do not at the study period before and after an SBHC is adopted. Shaded areas represent 95% confidence intervals. If the 95% CIs cross zero at every pre-period time point, we concluded that the counterfactual assumption of DID (i.e., the parallel pre-trends) is satisfied. Regression models are linear probability models with SBHCs adopted between 2007-2019 as the exposure; covariates included are the student characteristics, including sex, racial/ethnic identity, enrollment in special education, immigration status, housing stability, English as a second language, and economic disadvantage. Standard errors are clustered at the school level in all models
Abbreviations: SBHC = school-based health center

Year relative to SBHC adoption

**eFigure 5.** Event studies for healthcare use

Panel A: ED visits for mental health reasons

**
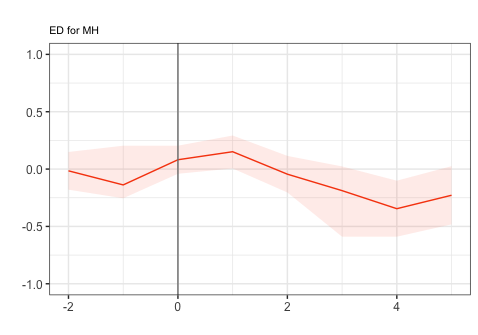
**

Percentage point difference
 in diagnoses

Panel B. ED visits for suicidal thoughts/behaviors


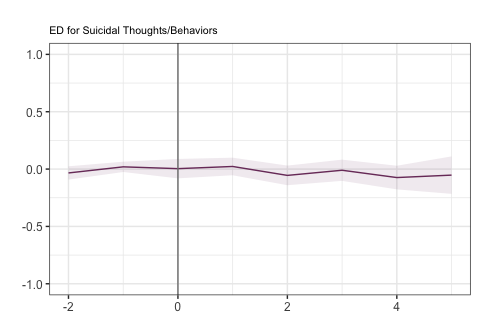


Percentage point difference
 in diagnoses

Year relative to SBHC adoption

Panel C. Outpatient mental health visits


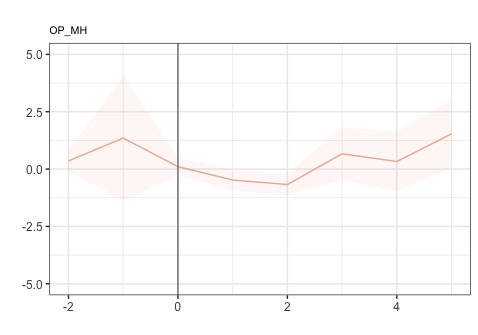


Percentage point difference
 in diagnoses

Panel D. Telemental health visits


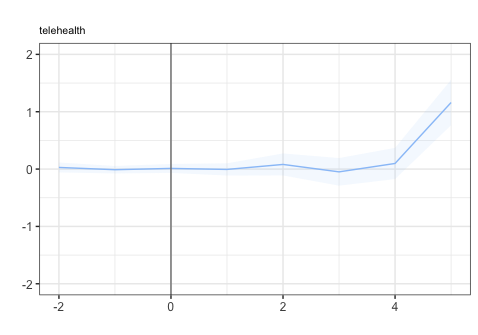


Percentage point difference
 in diagnoses

Year relative to SBHC adoption

Panel E. Psychotherapy visits


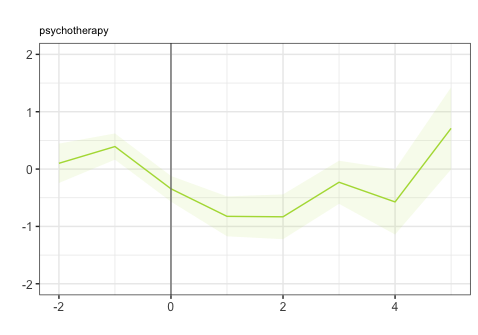


Percentage point difference
 in diagnoses

Panel F. Medications for mental health


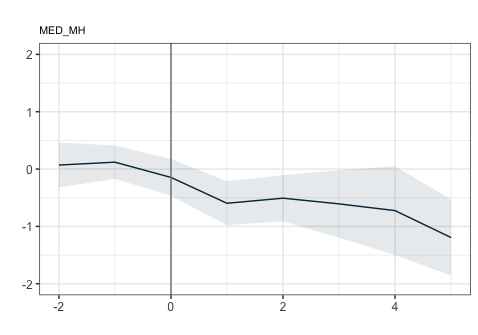


Year relative to SBHC adoption

Percentage point difference
 in diagnoses

Panel G. School-based visits


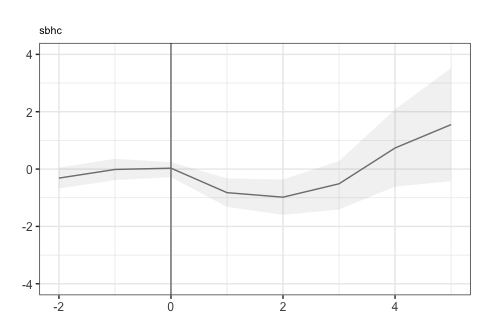


Percentage point difference
 in diagnoses

SOURCE/NOTES **Source** Authors’ analyses of linked Medicaid claims and administrative education data in Tennessee between 2006-2021. **Notes** Lines represent the differential estimates between from the districts that adopt and SBHC and those that do not at the study period before and after an SBHC is adopted. Shaded areas represent 95% confidence intervals. If the 95% CIs cross zero at every pre-period time point, we concluded that the counterfactual assumption of DID (i.e., the parallel pre-trends) is satisfied. Regression models are linear probability models with SBHCs adopted between 2007-2019 as the exposure; covariates included are the student characteristics, including sex, racial/ethnic identity, enrollment in special education, immigration status, housing stability, English as a second language, and economic disadvantage. Standard errors are clustered at the school level in all models
Abbreviations: SBHC = school-based health center

**eTable 7.** Results of sensitivity analyses for difference-in-differences

|  | **Stacked DID**  2007-2021 *N=25,436* | **Stacked DID**  2007-2019 *N=10,907* | **Staggered DID (C&S)**  2007-2019 *N=21,947* |
| --- | --- | --- | --- |
| *Mental Health Diagnoses* |  |  |  |
| Any mental health diagnosis | -0.76** (0.25) | -0.96** (0.33) | -0.56** (0.20) |
| Anxiety | -0.47*** (0.11) | -0.22 (0.14) | -0.001 (0.13) |
| ADHD | -0.50* (0.22) | -0.99** (0.30) | -0.50* (0.21) |
| Depression | -0.45** (0.13) | -0.19  (0.16) | 0.07 (0.14) |
| Suicidal thoughts and behaviors | -0.003  (0.03) | 0.04 (0.03) | -0.01 (0.05) |
| *Healthcare use* |  |  |  |
| ED visit for mental health | -0.15* (0.07) | -0.33*** (0.09) | -0.12 (0.07) |
| ED visit for suicidal thoughts/behaviors | -0.01 (0.03) | 0.05 (0.03) | -0.04 (0.06) |
| Outpatient mental health visit | 0.95** (0.28) | 0.43 (0.40) | 0.46 (0.37) |
| Telemental health visit | -0.36*** (0.04) | -0.18** (0.05) | 0.55*** (0.1) |
| Psychotherapy | 0.31 (0.18) | -0.07 (0.24) | -0.22 (0.16) |
| MH medication use | -0.69** (0.22) | -1.07** (0.3) | -0.62** (0.20) |
| School-based visit | 0.91** (0.33) | 1.64*** (0.36) | 0.11 (0.38) |

***p<0.001; **p<0.01; *p<0.05

SOURCE/NOTES **Source** Authors’ analyses of linked Medicaid claims and administrative education data in Tennessee between 2006-2021. **Notes** Estimates are the group-time average treatment effects from each of the specified estimators. Regression models are linear probability models with SBHCs adopted between 2007-2019 as the exposure; covariates included are the student characteristics, including sex, racial/ethnic identity, enrollment in special education, immigration status, English as a second language, and economic disadvantage. Standard errors are clustered at the school level in all models
Abbreviations: SBHC = school-based health center; ED = emergency department
